# Gut microbiota from mice with cerebral ischemia-reperfusion injury affects the brain in healthy mice

**DOI:** 10.18632/aging.202763

**Published:** 2021-03-26

**Authors:** Hongru Wang, Shangjun Ren, Hailing Lv, Lili Cao

**Affiliations:** 1 Department of Neurology, Qilu Hospital, Cheeloo College of Medicine, Shandong University, Jinan 250012, Shandong, China; 2 Department of Neurology, Liaocheng People’s Hospital, Liaocheng 252000, Shandong, China; 3 Department of Neurosurgery, Liaocheng People’s Hospital, Liaocheng 252000, Shandong, China; 4 Department of Neurology, Shandong Provincial Third Hospital, Cheeloo College of Medicine, Shandong University, Jinan 250000, Shandong, China

**Keywords:** gut microbiota, cerebral ischemia-reperfusion, microbiota colonization, functional connectivity, hippocampus

## Abstract

Gut microorganisms can profoundly influence brain function in the host and their behavior. Since altered brain functional connectivity (FC) has been implicated in various cerebrovascular disorders, including cerebral ischemia-reperfusion (I/R) injury, we hypothesized that gut microbiota in mice with cerebral I/R injury would affect brain FC when transplanted into germ-free mice. Metagenomic analysis of germ-free male C57BL/6J mice colonized with microbiota from mice with and without cerebral I/R injury showed a clear distinction in microbiota composition between mice colonized with control and I/R microbiota. The I/R microbiota-colonized mice showed decreased FC in the cingulate cortex, hippocampus, and thalamus, and exhibited increased anxiety as well as diminished spatial learning and memory and short-term object recognition memory. I/R microbiota-colonized mice also had significantly reduced dendritic spine density and synaptic protein levels and exhibited increased hippocampal inflammation. These results indicate that gut microbiota components from mice with cerebral I/R injury can alter animal behavior, brain functional connectivity, hippocampal neuronal plasticity, and neuroinflammation. Moreover, they increase our understanding of the mechanisms through which the gut microbiome contributes to the pathobiology of cerebrovascular diseases.

## INTRODUCTION

The gut microbiome and the human host have an intimate and bidirectional symbiotic interaction [1]. The gut has been denoted as the second brain since it shares some comparable functions that affect mental, emotional, and cognitive well-being [2–4]. Hence, the gut-brain axis is a bidirectional communication pathway between the microbiota, gut, and central nervous system. The microflora in the intestinal tract plays a significant role in host physiology has been established [5]. Changes in microbiota composition can influence host behavior and have been linked to neuropsychiatric disorders as well as other diseases [6]. Cerebral infarction induced by middle cerebral artery occlusion leads to a persistent host intestinal microbiota dysbiosis, mucosal damage, and chronic systemic inflammation in cynomolgus monkeys [6]. In a mouse model of cerebral hypoperfusion, the microbiota has an essential role in spatial learning and memory impairment [7]. In germ-free animals, decreased resting-state functional MRI-based connectivity is observed, in addition to altered expression of synaptic plasticity-related genes, and hyper-neuroinflammation [8–10]. Emerging data suggested disconcertion in microbiota composition affected behaviors including anxiety, cognition, nociception, and social interaction, among others [11]. Dysbiosis of microbiota can influence the brain-neural function, possibly resulting in a further deterioration after stroke [12].

Since microbiota may regulate behavior [13–15], intestinal bacteria might play a crucial role in regulating the development, expression, and outcomes of numerous cerebrovascular diseases [16]. A previous meta-analysis has indicated a positive dose-dependent association between gut microbe-generated metabolites and increased stroke risk and mortality [17]. For cerebral ischemia-reperfusion (I/R) injury, several studies have shown differences in microbiota composition between patients with stroke and health. Stroke was related to perturbations in microbiota composition and more opportunistic pathogens, including *Enterobacter, Megasphaera, Oscillibacter,* and *Desulfovibrio*, while commensal or beneficial genera (*Bacteroides, Prevotella,* and *Faecalibacterium*) are fewer abundant [18]. Additionally, systemic exposure to *Porphyromonas gingivalis* has been related to an increased possibility of ischemic stroke [19]. Continual Gram-negative bacteria venous injection promotes stroke in stroke-prone spontaneously hypertensive rats [20]. Antibiotic-induced alterations in the intestinal microbiota lessen ischemic brain injury in mice [21]. Furthermore, stroke outcomes can be influenced by the composition of the enteric microbiome [22].

In this study, we investigated the impact of microbiota from mice with cerebral ischemia on the brain functional connectivity and behavior of healthy mice. We hypothesized that enteric microbiota of mice with repeated global cerebral ischemia induced by bilateral common carotid arteries (BCCAO) would modify brain functional connectivity, behavior, hippocampal neuronal plasticity, and neuroinflammation when transplanted into germ-free mice of the same age. To investigate this hypothesis, we colonized germ-free mice with enteric microbiome assembled from male BCCAO mice (mice^BCCAO^) or age-matched healthy sham-operated mice (mice^control^), and carried out resting-state functional magnetic resonance imaging (rs-fMRI) and behavior analysis. Elucidating the effects of the BCCAO microbiome on the brain and behavior will lead to a better understanding of cerebral I/R injury, and the development of novel therapeutic methods for cerebrovascular diseases by targeting the microbiota.

## RESULTS

### Differences in microbiota composition between mice^control^ and mice^BCCAO^

**Figure 1:**
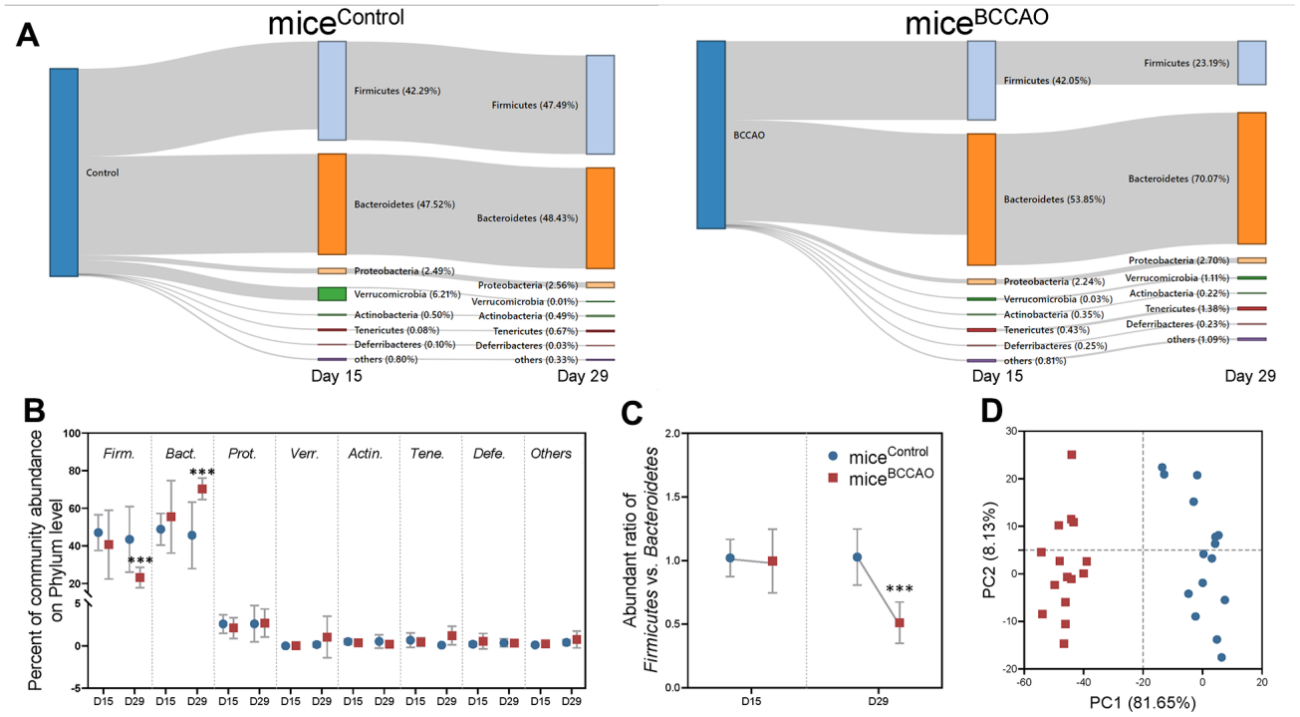
**Microbial analyses on the phyla level.** (**A**) Sankey graph showing changes in gut microbial composition on the 15th and 29th day after fecal microbiota transplantation. (**B**) Percent of community abundance on the phyla level. *Firm*. means *Firmicutes*, *Bact*. means *Bacteroidetes*, *Prot*. means *Proteobacteria*, *Verr*. means *Verrucomicrobia*, *Acti*. means *Actinobacteria*, *Tene*. means *Tenericutes*, *Defe*. means *Deferribacteres*. (**C**) Ratio of *Firmicutes* and *Bacteroidetes* on the 15th and 29th days. (**D**) Principal component analysis (PCA) plot of weighted UniFrac distances showing a clear separation in microbial composition between mice^control^ and mice^BCCAO^. *** denotes *P* < 0.001 compared with control mice using a two-way repeated-measures ANOVA with post-hoc Tukey multiple comparisons test. All values are expressed as means ± S.D; n=15.

**Figure 2:**
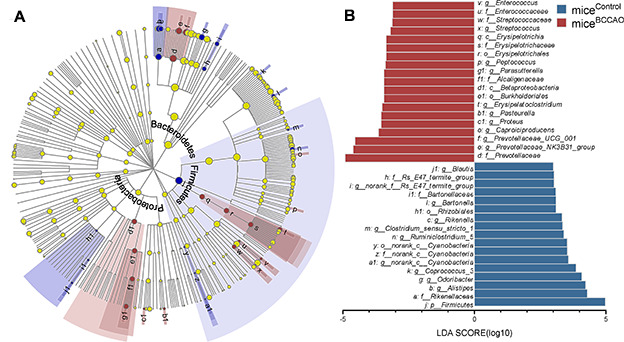
**Microbial analyses.** (**A**) The central point represents the root of the tree (bacteria), and each ring represents the next lower taxonomic level (phylum to genus). The diameter of each circle represents the relative abundance of the taxon. (**B**) The different bacterial genera between mice^control^ and mice^BCCAO^ using linear discriminant analysis effect size (LEfSe) analysis, linear discriminant analysis (LDA) score > 3.0, *P* < 0.05 unadjusted; n=15.

First, we assessed the microbiota composition of feces from mice^control^ and mice^BCCAO^. We performed a metagenomic analysis to reveal the gut microbiota compositions at the phylum and genus levels in both groups. 15 days after fecal microbiota transplantation, the gut microbial composition in mice^control^ and mice^BCCAO^ was 47.52% vs. 53.85% *Bacteroidetes*, 42.29% vs. 42.05% *Firmicutes*, 2.49% vs. 2.24% *Proteobacteria*, 6.21% vs. 0.03% *Verrucomicrobia*, 0.50% vs. 0.35% *Actinobacteria*, 0.08% vs. 0.43% *Tenericutes*, 0.10% vs. 0.25% *Deferribacteres*, and 0.80% vs. 0.81% other phyla. After 29 days, there were 48.43% vs. 70.07% *Bacteroidetes*, 47.49% vs. 23.19% *Firmicutes*, 2.56% vs. 2.70% *Proteobacteria*, 0.01% vs. 1.11% *Verrucomicrobia*, 0.49% vs. 0.22% *Actinobacteria*, 0.67% vs. 1.38% *Tenericutes*, 0.03% vs. 0.23% *Deferribacteres*, and 0.33% vs. 1.09% other phyla in the gut of mice^control^ and mice^BCCAO^ (Figure 1A). The relative abundance of *Firmicutes* significantly decreased, while the percentage of *Bacteroidetes* significantly increased in mice^BCCAO^ compared to mice^control^ 29 days after transplantation (Figure 1B). Moreover, mice^BCCAO^ showed a remarkably decreased ratio of *Firmicutes* to *Bacteroidetes* on the 29th day (Figure 1C). We performed a principal component analysis (PCA) to determine the influence of baicalin treatment on the gut microbial populations at the phylum level. The plot of principal component 1 (PC1) against PC2 showed that each group formed a distinct cluster; mice^BCCAO^ clustered separately from mice^control^ (Figure 1D). Linear discriminant analysis effect size (LEfSe) test was used to assess the microbiota alterations after BCCAO microbiota treatment. The structure and predominant microbiota in both groups were represented as a cladogram (Figure 2A). The LEfSe analysis showed that 20 genera differed in relative abundance between the two experimental groups with linear discriminant analysis (LDA) score > 3.0 and *P*_unadjusted_ < 0.05 (Figure 2B). These genera belonged to the phyla *Bacteroidetes* (7/20), *Firmicutes* (9/20), *Proteobacteria* (3/20), and *Cyanobacteria* (1/20). In total, 10 genera were enriched in mice^BCCAO^, while 10 other genera were more abundant in mice^control^. From the genera enriched in mice^BCCAO^, 3 belonged to the family of *Prevotellaceae* within the phylum *Bacteroidetes* (including *Prevotellaceae_UCG_001*, *Prevotellaceae_NK3B31_group*, *Pasteurella*), 2 belonged to the order of *Lactobacillales* (including *Enterococcus* and *Streptococcus*), and 2 belonged to the order of *Clostridiales* (including *Peptococcus* and *Caproiciproducens*) within the phylum *Firmicutes*. These results indicated that the BCCAO microbiota colonization negatively impacted the mice's gut microbiota.

### Decreased functional connectivity in mice^BCCAO^

**Figure 3:**
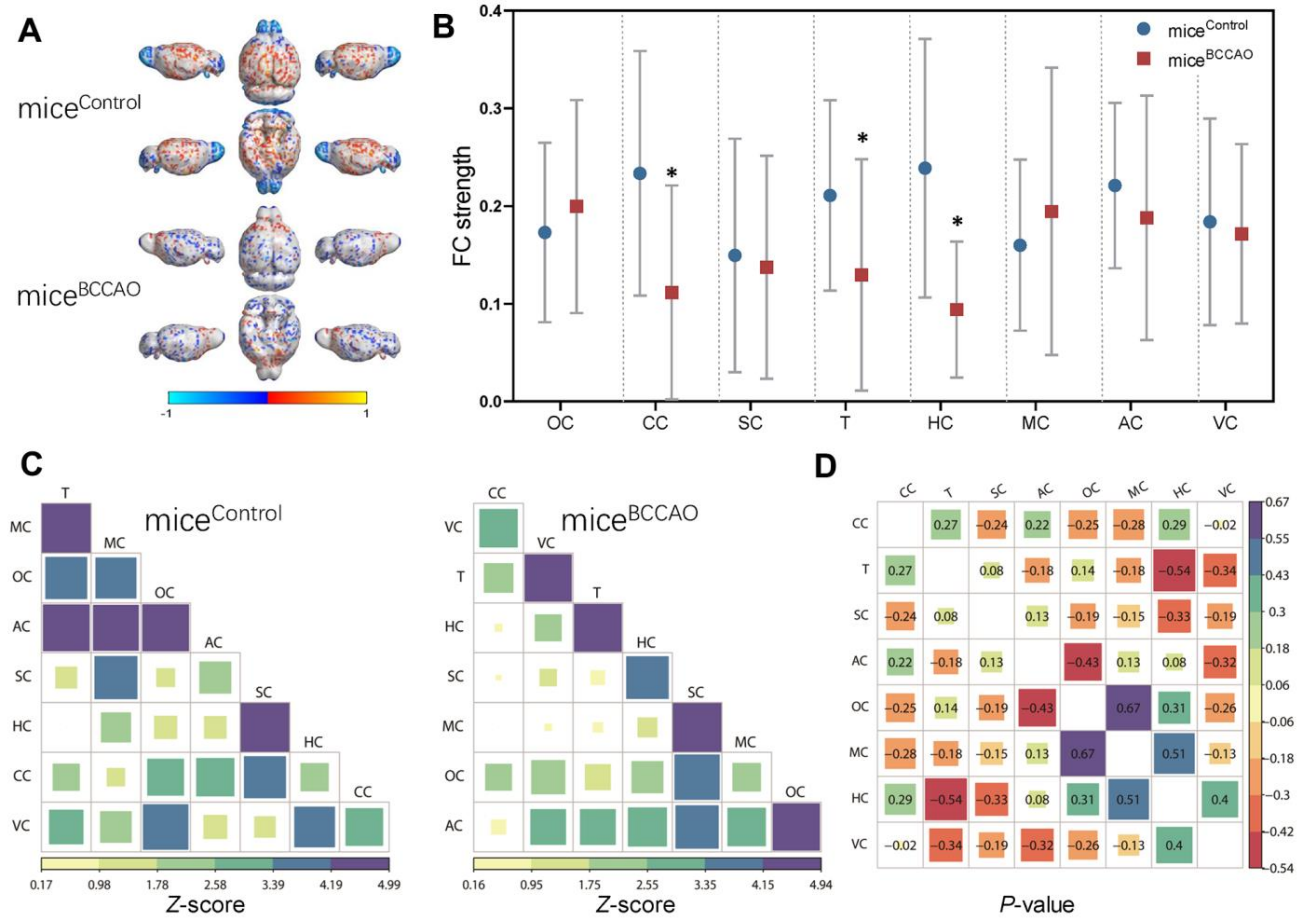
**Effect of BCCAO microbiota on brain structure and function.** (**A**) Virtual graphics show brain functional connectivity (FC) in mice^control^ and mice^BCCAO^. (**B**) The mean functional connectivity strength per brain network. * denotes *P* < 0.05 compared with control mice using a two-way repeated-measures ANOVA with post-hoc Tukey multiple comparisons test. All values are expressed as means ± S.D; n=15. (**C**) The mean FC matrices show the strength of functional connectivity between pairs of brain regions in control and BCCAO-treated groups. The color scale represents the strength of functional connectivity. (**D**) Correlation analyses of 8 regions of interest (ROIs) in the mouse brain. The brain regions analyzed include orbitofrontal cortex (OC), cingulate cortex (CC), somatosensory cortex (SC), thalamus (T), hippocampus (HC), motor cortex (MC), auditory cortex (AC), and visual cortex (VC).

Next, we compared the brain ROIs based on rs-fMRI to evaluate the effect of BCCAO microbiota colonization on the brain FC patterns. The ROIs were chosen based on the results of rs-fMRI and included auditory cortex (AC), cingulate cortex (CC), hippocampus (HC), motor cortex (MC), orbitofrontal cortex (OC), somatosensory cortex (SC), thalamus (T), and visual cortex (VC). The functional connections between these ROIs are shown in the virtual graphs (Figure 3A). The results show decreased FC in the cingulate cortex, hippocampus, and thalamus of mice^BCCAO^ compared to control mice (Figure 3B). We found no changes in FC correlation analysis between each ROIs in the correlation matrices (Figure 3C, 3D). These data indicated that BCCAO microbiota colonization decreased the brain FC in mice^BCCAO^.

### Increased anxiety and memory deficits in mice colonized with BCCAO microbiota

**Figure 4:**
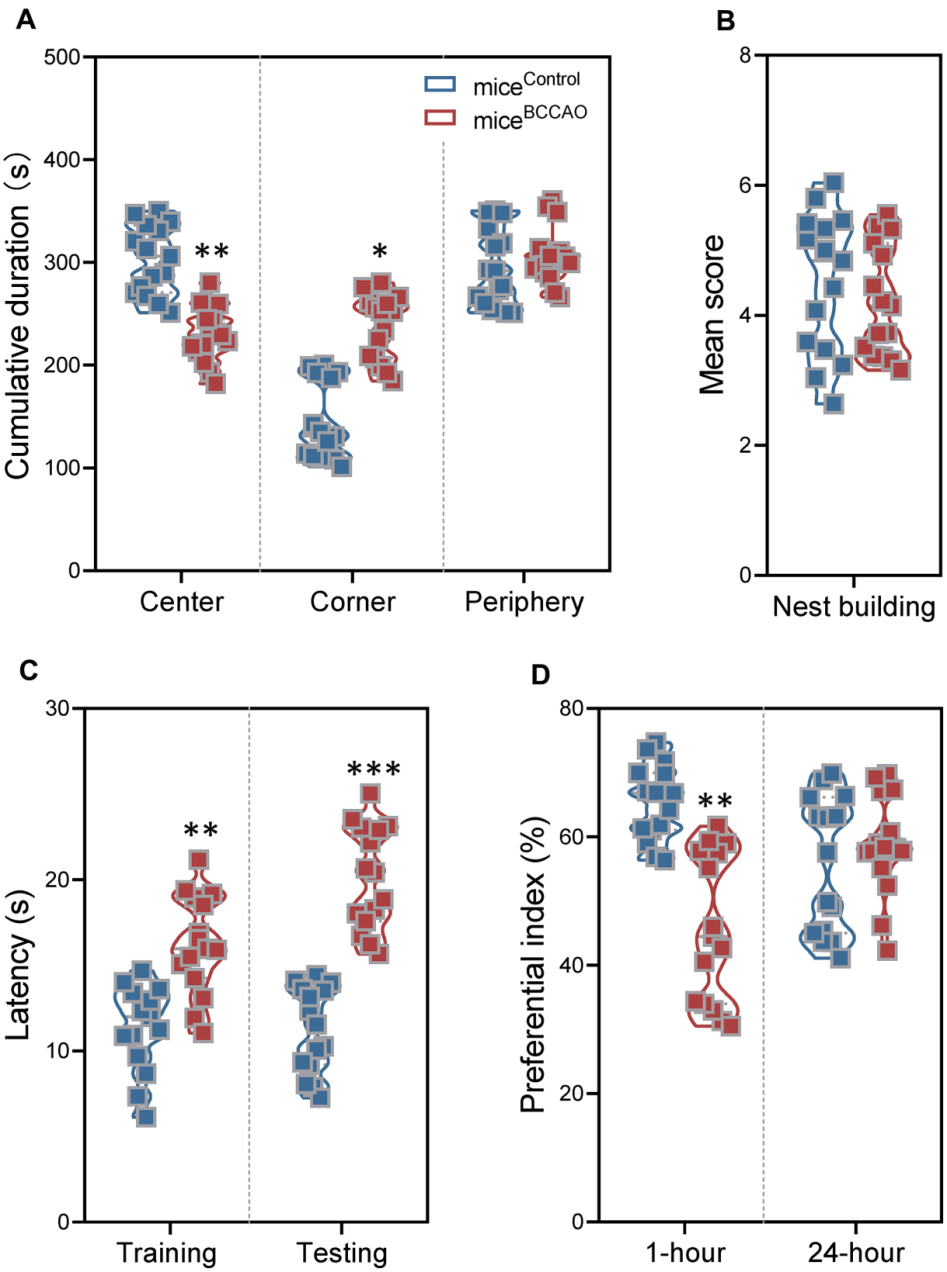
**Effect of BCCAO microbiota on mice behavior.** (**A**) Time spent in the center (left), corner (middle), and periphery (right) of the open field test. (**B**) The nest-building scores of mice^control^ and mice^BCCAO^. (**C**) The latency in the training and testing phases for mice^control^ and mice^BCCAO^ in the Morris water maze test. (**D**) The preferential index (%) after 1 hour (left) and 24 hours (right) of training in the testing phase of the novel object recognition test. * denotes *P* < 0.05, ** denotes *P* < 0.01 and *** denotes *P* < 0.001 compared with control mice using unpaired Student`s *t*-tests or a two-way repeated-measures ANOVA with post-hoc Tukey multiple comparisons test. All values are expressed as means ± S.D, n=15.

Next, we explored the effect of mouse BCCAO microbiota on the behavior of healthy mice. In the open field test (OFT), we assessed the spontaneous locomotor activity. Compared to control mice, mice^BCCAO^ spent more time in the corners, and less time in the center (Figure 4A). There was no difference in the time spent in the periphery arena. The increased time spent in the corners in mice^BCCAO^ indicated increased anxiety. The crunching of harmless objects, such as cotton or soft papers to build a nest, has been associated with compulsive or natural behavior. However, the nest building test did not show any differences in nest building scores between mice^control^ and mice^BCCAO^ (Figure 4B).

To examine spatial learning and memory, and object recognition memory, we performed the Morris water maze (MWM) test and the novel object recognition (NOR) test. During the training and testing phases of the MWM test, mice^BCCAO^ showed significantly longer escape latencies compared to control mice (Figure 4C). Besides, mice^BCCAO^ demonstrated a significantly lower preferential index compared to mice^control^ in the NOR test using the 1-hour interval between training and testing phases; however, there was no difference when the interval between training and testing phases was 24 hours (Figure 4D). These findings showed that oral treatment with BCCAO microbiota declined spatial learning and memory, and decreased short-term object recognition memory in mice.

### Decreased hippocampal neuronal plasticity in BCCAO microbiota-colonized mice

**Figure 5:**
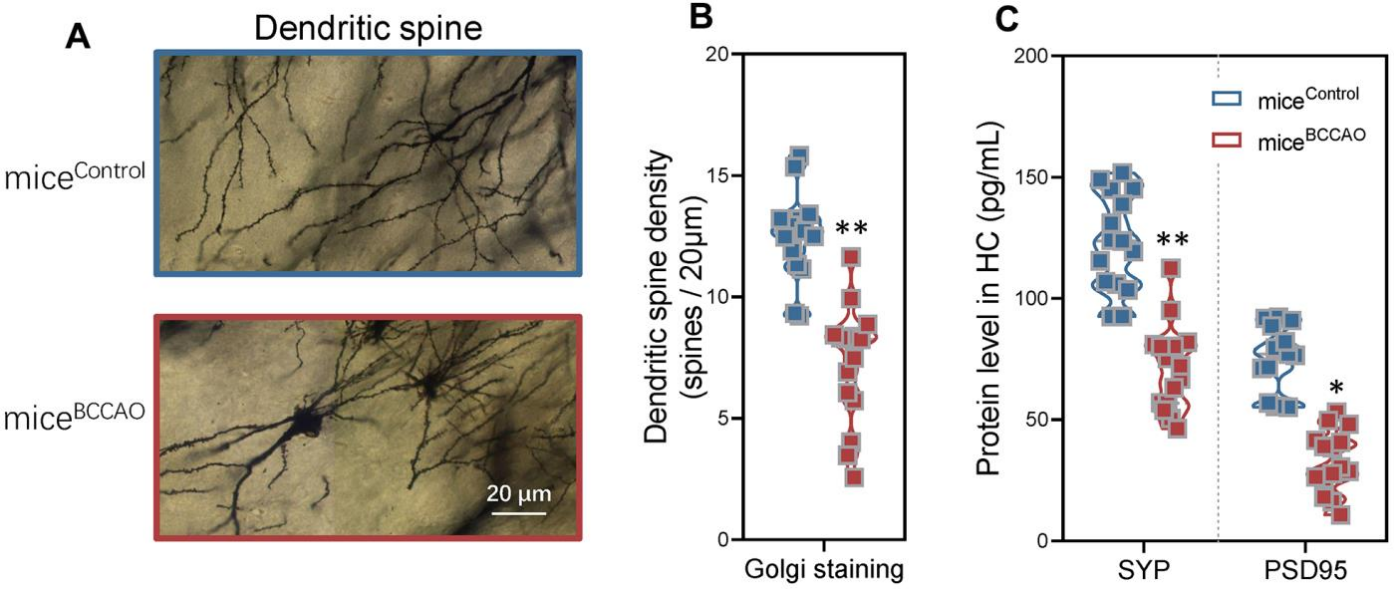
**Effect of BCCAO microbiota on hippocampal neuronal plasticity.** (**A**) Representative confocal microscopic images of the Golgi-stained hippocampal regions in mice^control^ and mice^BCCAO^. (**B**) Mean dendritic spine densities of the hippocampal neurons based on the analysis of Golgi-stained brain tissue sections of mice^control^ and mice^BCCAO^. (**C**) Protein levels of synaptophysin (SYP) and PSD95 in the hippocampal region. *denotes *P* < 0.05 and ** denotes *P* < 0.01 compared to control mice using unpaired Student`s *t*-tests. All values are expressed as means ± S.D; n=15.

To investigate the effect of BCCAO microbiota colonization on hippocampal neuronal plasticity, we assessed dendritic spine density with the Golgi staining. Golgi staining displayed neuropathological modifications in the hippocampal region in mice^BCCAO^ (Figure 5A), and indicated a significant decrease in the dendritic spine density in the hippocampus of BCCAO microbiota-colonized mice (Figure 5B). Moreover, the levels of synaptophysin (SYP) and PSD95 were significantly decreased in the hippocampal region in mice^BCCAO^ (Figure 5C).

### BCCAO microbiota colonization increases hippocampal neuroinflammation

**Figure 6:**
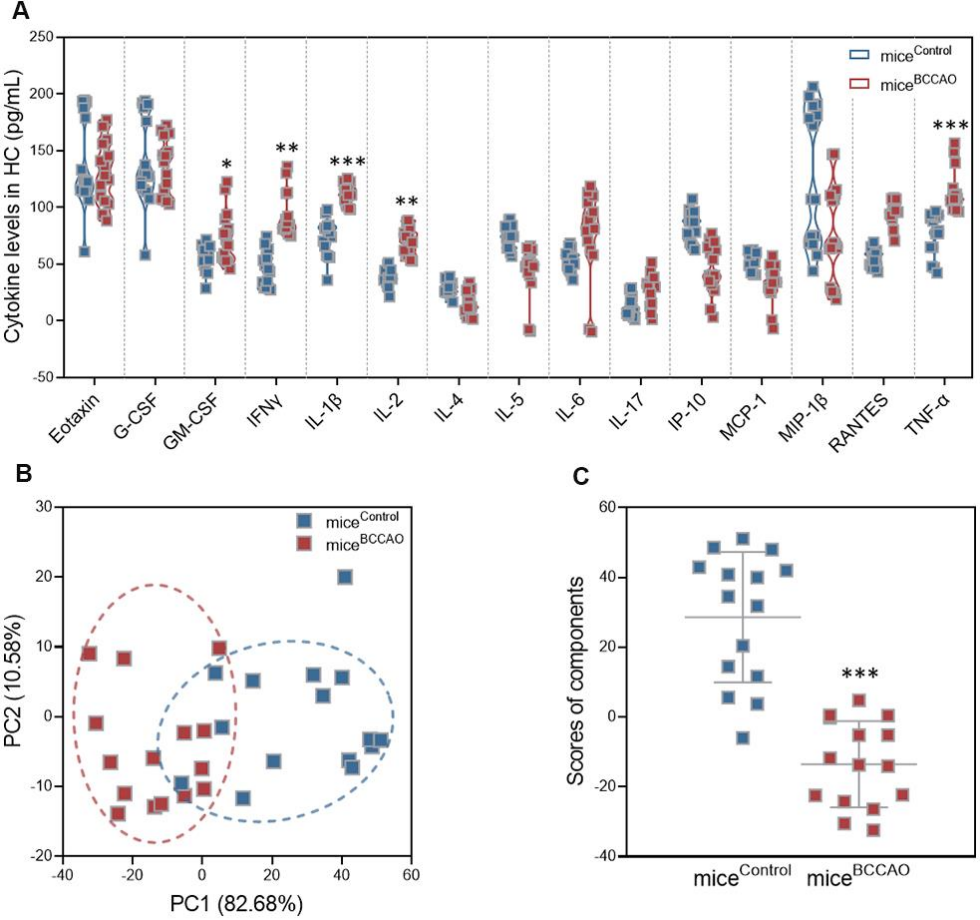
**Effect of BCCAO microbiota on hippocampal neuroinflammation.** (**A**) Levels of G-CSF, GM-CSF, IL-1β, IL-2, IL-4, IL-5, IL-6, IL-17, IFNγ, IP-10, TNF-α, RANTES, MCP-1, eotaxin, and MIP-1β in the hippocampus of mice^control^ and mice^BCCAO^. (**B**) PCA of the data from multiplex bead analysis; each dot represents one mouse. (**C**) Mean PCA scores of mice with and without BCCAO microbiota. *, *P* < 0.05; **, *P* < 0.01; and ***, *P* < 0.001 compared with control mice using unpaired Student`s *t*-tests or a two-way repeated-measures ANOVA with post-hoc Tukey multiple comparisons test. All values are expressed as means ± S.D; n=15.

Because of the observed differences in hippocampal neuronal plasticity, we next assessed the levels of pro-inflammatory cytokines G-CSF, GM-CSF, IL-1β, IL-2, IL-4, IL-5, IL-6, IL-17, IFNγ, IP-10, TNF-α, RANTES, MCP-1, eotaxin, and MIP-1β in the hippocampus by multiplex bead analysis. Mice^BCCAO^ had significantly increased levels of pro-inflammatory cytokines, including GM-CSF, IFNγ, IL-1β, IL-2, and TNF-α (Figure 6A). Principal component analysis (PCA) revealed that PC1 and PC2 grouped mice formed two distinct clusters (Figure 6B). To further investigate the effect of BCCAO microbiota colonization on neuroinflammation, average PCA scores were graphically illustrated (Figure 6C). This illustration revealed that the average neuroinflammation score was significantly different between mice^control^ and mice^BCCAO^, indicating increased neuroinflammation in mice^BCCAO^.

### Changes in microbiota are associated with behavioral and neurobiological alterations

**Figure 7:**
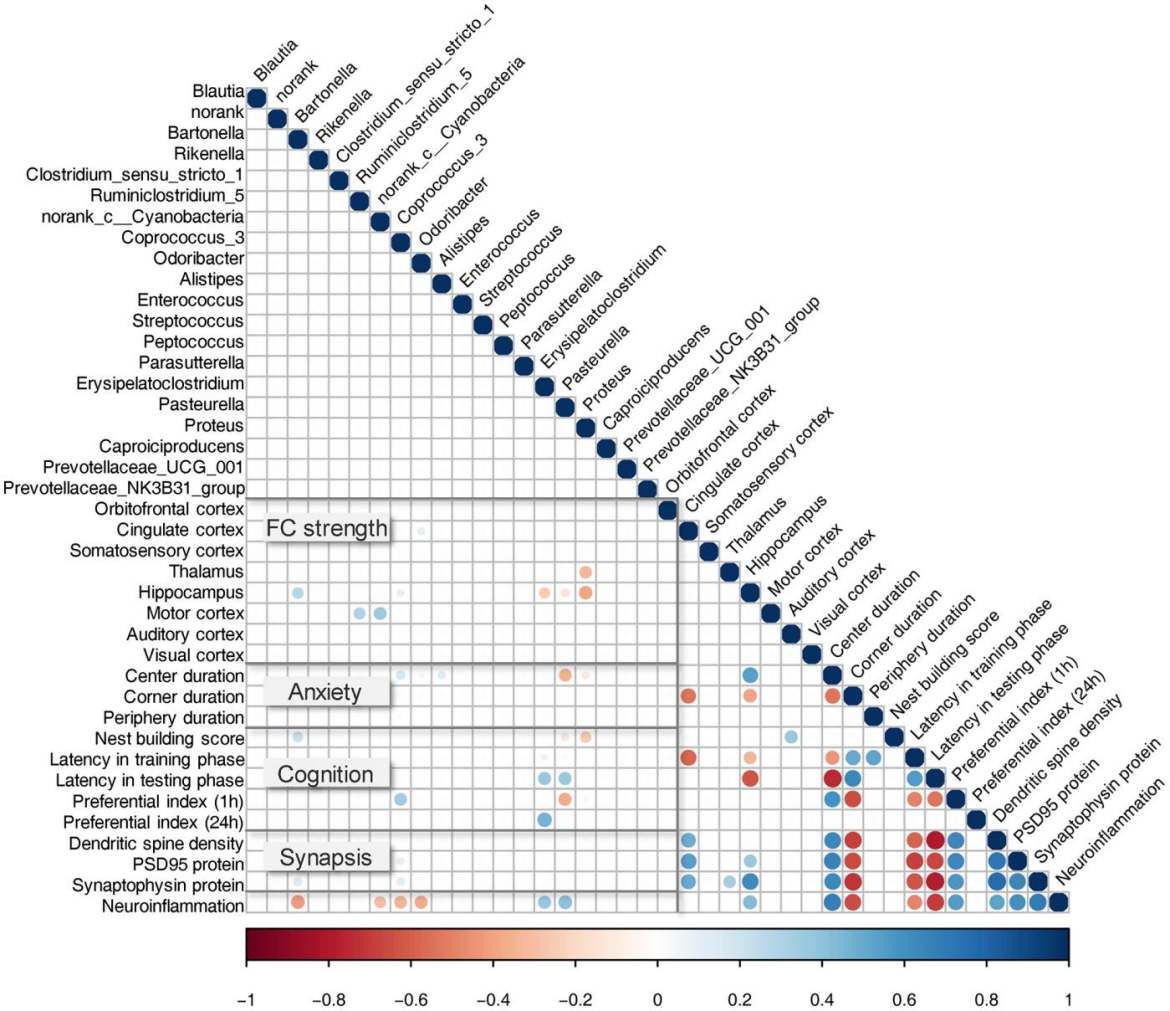
**Correlation between bacterial genera and key behavioral/neurobiological findings.** Pearson correlation between relative taxa abundance and cognitive behavior test, fMRI measured FC, hippocampal neuronal plasticity, and neuroinflammation. All observed correlations were statistically significant (*P* < 0.01); strong correlations are indicated by large circles, and weaker correlations by small circles. The colors signify whether the correlation was negative (red) or positive (blue).

We investigated whether the microbiota changes described above (Figure 2) correlated with changes in behavioral and neurobiological characteristics, such as anxiety, cognition, fMRI measured FC, hippocampal neuronal plasticity, and neuroinflammation. The results are illustrated in Figure 7 (*P* < 0.01). FC strength of the cingulate cortex showed a positive correlation with the relative abundance of *Odoribacter*. Thalamus showed a negative correlation with the relative abundance of *Proteus*. Hippocampus showed a positive correlation with the relative abundance of *Bartonella*, *Coprococcus_3*, and a negative correlation with *Erysipelatoclostridium*, *Pasteurella*, and *Proteus*. The motor cortex showed a negative correlation with *Ruminiclostridium_5* and *norank_c_Cyanobacteria*. In anxiety-like behavior, the time spent in the center of the open field showed a positive correlation with *Coprococcus_3*, *Enterococcus*, and a negative correlation with *Pasteurella* and *Proteus*. In cognitive behavior, nest building ability showed a positive correlation with *Bartonella*, and a negative correlation with *Caproiciproducens* and *Proteus*. Latency in the testing phase of the MWM test showed a positive correlation with *Pasteurella* and *Proteus*. Preferential index with 1-hour interval between training and testing phase showed a positive correlation with *Coprococcus_3*, and a negative correlation with *Pasteurella*. The preferential index (24 h) showed a positive correlation with *Erysipelatoclostridium*. In hippocampal neuronal plasticity, the levels of PSD95 protein showed a positive correlation with *Coprococcus_3*. The SYP protein showed a positive correlation with *Bartonella* and *Coprococcus_3*. The neuroinflammation status in the hippocampus showed a negative correlation with *Bartonella*, *norank_c_Cyanobacteria*, *Coprococcus_3*, and *Odoribacter*, and a positive correlation with *Erysipelatoclostridium* and *Pasteurella*.

## DISCUSSION

Based on the recently reported association between gut microbiota and neurodevelopmental dysfunction by the gut-brain axis [23, 24], we hypothesized that the microbiota from mice with repeated global cerebral ischemia-induced by BCCAO would modify brain functional connectivity (FC), behavior, hippocampal neuronal plasticity, and neuroinflammation when transplanted into germ-free mice of the same age. Our results demonstrated that the BCCAO microbiota-colonized mice had a decreased FC strength of the cingulate cortex, hippocampus, and thalamus.

Increasing evidence implicates the gut microflora as a crucial regulator of brain function and behavior changes. Gut dysbiosis induced by end-stage renal disease has been associated with cingulate cortex based functional connectivity pattern [25]. Probiotics supplementation has been shown to modify resting-state neural oscillations, with an increased theta and alpha band power in the cingulate cortex [26]. Functional neuroimaging studies in healthy humans have suggested that the cingulate cortex plays an important role in cognitive control [27]. The mean fractional anisotropy of the cingulate cortex correlates with processing speed, working memory, and executive attention [28]. Ischemia has been shown to activate astrocytes in the cingulated cortex as reflected by elongated dendrites, astrocyte stomata hypertrophy, and immunoreactivity of glial fibrillary acidic protein [29]. Another affected FC of the brain region in our study was the hippocampus. The hippocampus is extremely sensitive to senescence, life experiences, and environmental factors [30–32]. The hippocampus is vital for response inhibition, episodic memory, spatial cognition, neural plasticity, and mood regulation. It is therefore not surprising that deteriorated hippocampal function has been implicated in many neurodevelopmental and psychiatric disorders. The third altered brain region in our study was the thalamus. Thalamus has multiple functions that regulate consciousness, sleep, and alertness [33]. The relative abundance of gut bacterial phyla has been linked to magnetic resonance imaging diffusion tensor imaging variables in the thalamus [34]. Our rs-fMRI data revealed decreased FC strength in the cingulate cortex, hippocampus, and thalamus in BCCAO microbiota-colonized mice. A disturbed FC strength was reported in consecutive reperfused stroke patients [35]. Moreover, there was a positive correlation between functional connectivity and functional outcome in patients.

Using the OFT test, we found that mice^BCCAO^ displayed more anxiety than mice^control^. However, the anxiety-like behavior is not specific to repeated cerebral I/R injury, since many psychiatric and neurologic disorders, including Alzheimer's disease, Parkinson's disease, and schizophrenia are comorbid with anxiety [36, 37]. Altered anxiety-like behavior has been also found after microbiota operations, and fecal transplantation [38]. These findings indicate that components of the intestinal flora can regulate anxiety. We found no differences in compulsive or natural behavior between BCCAO and control mice using the NBT test. Besides, global cerebral ischemia followed by reperfusion frequently results in significant cognitive learning and memory deficits [39]. Spatial learning and memory were assessed by the time required to find the platform or removed platform in the MWM test. The short-term and long-term object recognition memory was assessed by the preferential index in the NOR test. We found that the BCCAO mice displayed increased latency and decreased preferential index, which might be correlated with the decreased FC caused by microbiota transplantation.

Previous studies have indicated differences in the microbiota composition in individuals and mice with cerebral ischemia [40, 41]; however, transplantation of the microbiota from mice with ischemia into germ-free mice is novel. Besides, previous studies have shown that stroke lesions affected gut microbiota maladjustment by two different models of acute middle cerebral artery occlusion [12, 42]. In our study, analysis of the global microbial composition on the phyla level revealed a clear difference between microbiota from mice^BCCAO^ and mice^control^ (Figure 1D). We observed a significantly decreased ratio of *Firmicutes* to *Bacteroidetes* in mice^BCCAO^. The ratio of *Firmicutes* to *Bacteroidetes* is regarded to be highly significant in the gut microbiota status [43]. Multiple studies have shown that the decreased *Firmicutes*/*Bacteroidetes* ratio correlates with alcoholic liver, obesity, stroke, and other diseases [44–46]. Analyzing the global microbial composition on the genera level, we found an alteration in microbial composition, with 20 genera exhibiting significantly different relative abundance between the experimental groups by LDA. One of the different families between the two groups was *Prevotellaceae*, which was also decreased in an experimental stroke model and correlated with the extent of brain injury [47]. The relative abundance of *Enterococcus* and *Streptococcus* was also affected by cerebral ischemic stroke [48, 49]. Besides, we found that *Pasteurella, Peptococcus,* and *Caproiciproducens* were enriched in mice^BCCAO^; this was not reported before. Other significantly affected genera in this study belonged predominantly to the phylum *Firmicutes* (9/20), and families *Lachnospiraceae* and *Rikenellaceae*, which were related to locomotor behavior, mental well-being, and memory function [11, 50]. Two genera of the *Lachnospiraceae* and two of *Rikenellaceae* were less abundant in mice^control^ compared to mice^BCCAO^.

Our correlation analysis revealed that there was a positive correlation between FC strength of the hippocampus and the relative abundance of *Bartonella* and *Coprococcus*, and a negative correlation with *Erysipelatoclostridium*, *Pasteurella*, and *Proteus*. The abundance of these bacteria correlated with other behavioral or neurobiological findings. *Bartonella* positively correlated with nest building ability and hippocampal SYP protein levels, and negatively correlated with the neuroinflammation status. *Coprococcus* positively correlated with short-term object recognition memory and hippocampal PSD95 protein levels. A previous study showed that the abundance of *Coprococcus* was decreased in diabetic fatty rats with psychological-stress-induced diabetes-associated cognitive decline [51]. *Erysipelatoclostridium* positively correlated with long-term object recognition memory. In a previous study, correlation analysis revealed that cognitive function correlated positively with *Muribaculum* and *Erysipelatoclostridium* abundance [12]. *Pasteurella* was negatively correlated with spatial learning and memory. The abundance of these genera was also altered in other disorders, including depression [52], allergic diseases [53], dopaminergic neurons, and motor functions [54]. Nevertheless, as far as we know, this is the first study that reports significant associations between these genera and brain FC strength alterations. Thus, it will be important to analyze this association in clinical populations of the cerebral I/R injury.

This study used a mouse model, rather than patients with repeated cerebral I/R injury. However, animal tissues might have a different distribution of taxonomic groups compared to human subjects with repeated cerebral I/R injury. Moreover, the choice of collecting feces from male mouse donors reduced the sex effects and non-controlled hormonal effects. In a controlled environment, the gut microbiota composition is regulated by sex hormones, both in humans and rodents [55, 56]; therefore, only the male mice samples were transplanted. Second, using germ-free mice allowed us to ensure that the mice were exposed only to microbes from the donor samples, and contained a defined microbial composition [57]. Nonetheless, germ-free mice showed numerous physiological differences compared to normally raised mice [58]. For instance, germ-free mice exhibited a decreased cognition and level of synaptogenesis genes [9, 59]. However, to be able to analyze the baseline brain and behavioral function characteristics, future studies might include another group of germ-free mice without any microbiota colonization.

In summary, our study showed that microbial components of the gut microbiota of mice with repeated global cerebral ischemia-induced by BCCAO were associated with modifications in brain functional connectivity, behavior, hippocampal neuronal plasticity, and neuroinflammation. While our data did not demonstrate that the cognitive impairments in cerebral I/R injury were triggered by alterations in bacterial composition, the observed brain alterations, albeit not specific to cerebral I/R injury, highlighted the importance of gut microflora, potentially through aberrant functional connectivity. Our findings contribute to our understanding of the cerebral I/R injury and might lead to the development of novel therapeutic strategies targeting the microbiome in cerebrovascular diseases.

## MATERIALS AND METHODS

### Mouse model of repeated cerebral ischemia-reperfusion

Forty-five male C57BL/6J mice (2-3 months of age) were received from the experimental animal center of Shandong University. Before BCCAO, the three or four mice per cage were group-housed in enriched, ventilated cages. The mice were randomly assigned to two groups, with the sham surgery group (n=20), BCCAO group (n=20). The model of repeated global cerebral ischemia was followed by reperfusion as previously described [60]. In short, mice were initially anesthetized with 4% isoflurane followed by intraperitoneal injections of 4 mg/kg xylazine and subsequently maintained with 1.5% isoflurane. The physique temperature was maintained at 37° C by a heating lamp. Bilateral carotid arteries were exposed by a midline incision on the anterior neck. Then, the common carotid arteries were isolated with 3-0 silk ligatures, and the arteries were occluded via microaneurysm clips for 30 min. The carotid artery was occluded two times, with a 10-minute interval between the two occlusions. The sham-operated mice acquired identical surgical procedures without ischemia.

### Germ-free mice

Thirty male, C57BL/6J mice wild-type mice received from Cyeagen Biosciences Inc (Jiangsu, China) and made germ-free via hysterectomy rederivation. The animals were housed in aseptic cages, under normal laboratory conditions, with one-way airflow, sterile food, and sterile water. The behavioral and electrophysiological experiments were performed in the gnotobiotic isolators. The mice were randomly divided into two therapy groups. Littermates were assigned to intervention before the experiment. The therapy groups were concealed from everyone involved. Three or four animals were kept in cages. The mice were tested in three separate series (Batch 1: mice^control^ n=6, mice^BCCAO^ n=4; Batch 2: mice^control^ n=5, mice^BCCAO^ n=5; Batch 3: mice^control^ n=4, mice^BCCAO^ n=6; total mice^control^: n=15, total mice^BCCAO^ n=15).

### Preparation of BCCAO mice fecal samples for colonization

The fecal implantation was carried out as described somewhere else with slight modifications [61]. Briefly, BCCAO mice fecal samples were placed on ice after collection and stored at -80° C until processing. The fecal samples of BCCAO mice were organized for colonization by way of pooling equal quantities of fecal material. A total of 100 mg samples was returned to 1 ml of 0.1M sterile PBS. Before centrifugation at 700g for 5 min, the solution was mixed steadily for 10 s by a vortex. The supernatant was accumulated as transplantation material and kept at -80° C.

### Experimental design

**Figure 8:**
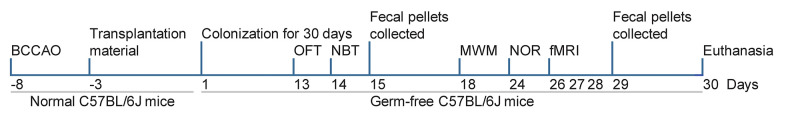
**Study design.** On day -8, the model of repeated global cerebral ischemia was induced by BCCAO. On day -3 to day 0, the BCCAO mice fecal samples for colonization were prepared. Germ-free mice were colonized with BCCAO mice or control mice microbiota on day 1 of the experiment. Fecal pellets were collected on days 15 and 29. The open-field test (OFT) was performed on day 13, the nest building test (NBT) on day 14, the Morris water maze test on days 18-23, and the novel object recognition test (NOR) on days 24 and 25. The mice underwent MRI on days 26, 27, or 28.

In total, forty male normal C57BL/6J mice (2-3 months of age) and 30 male germ-free C57BL/6J mice (1 month of age) were used in the study, blinded control. The study schedule is shown in Figure 8. The mouse model of repeated cerebral I/R injury was caused by BCCAO on day -8 of the test. The bacterial transplant material was organized from day -3 to 0. Germ-free mice were colonized with microbiota from BCCAO mice (mice^BCCAO^) or sham-operated mice (mice^control^) microbiota on day 1 via oral gavage for 30 days. The germ-free control group was colonized with gut microbiota from the mice^control^ group. On day 15 and 29, fresh fecal pellets were amassed between 9 a.m.to10 a.m., snap-frozen, and stored at -80° C till use. On day 26-28, MRI continuously scanned the animals in random order. On day 30, after the behavioral and MRI tests, mice were positioned in a closed chamber and euthanized by isoflurane inhalation and cervical dislocation to collect hemisphere, hippocampus, and plasma. All samples were saved in a 4% paraformaldehyde solution or at -80° C until use.

### Open field test

Animals were placed in the center of the open field (40 × 40 × 25 cm) with white walls and were free to explore the area for 15 min. The trial was videoed through a behavior monitoring program (XinRan Technology, China). The exploration activities in the corners (10 × 10 cm) and center (20 × 20 cm) were mechanically recorded. Between tests, the experimental amenities were cleaned and removed odor by 70% ethanol.

### Nest building test

The animals were kept in an individual and sterile testing container with one nestled (4 cm × 4 cm). The nesting scores were analyzed by three assessors the next morning, following a pre-determined scale (least: 1, best: 6).

### Morris-water maze test

The Morris water maze test was measured on day 18-23. In the water maze test, the animals were positioned in a white circular pool filled with 30-cm-depth opaque water. A platform of diameter 6 cm was submerged below 1 cm of water’s surface in the target quadrant. The training and testing phases were 60 s. In the training phase, the mice were placed into the water facing the wall of the pool at one of the four quadrants for five consecutive days. Each mouse was allowed 60 s to find the hidden platform. If the mouse did not find the platform, it was gently led onto the hidden platform for 5 s. In the testing phase, the hidden platform was removed and the mice explored for 60 s. The time to find the hidden platform in the training and testing phase was recorded and analyzed.

### Novel object recognition test

The test was performed on day 24 and 25. In general, each mouse was placed in the middle of an empty chamber (10 min) to be familiar with the testing condition. One-hour delay, mouse allowed to explore two same objects called A1 and A2 placed distantly from each other. After a 1-hour and 24-hour, a novel object (object B or C) was replaced by one of the objects A1. The preferential index $\text{(Preferential}\;\text{Index}=\frac{\text{exploration}\;\text{time}\;\text{for}\;\text{object}\;\text{B}\;\text{or}\;\text{C}}{\text{total}\;\text{amount}\;\text{of}\;\text{exploration}\;\text{of}\;\text{both}\;\text{objects}}\times 100)$ was calculated to measure non-spatial memory in 5 min phase.

### Magnetic resonance imaging (MRI)

All MRI measurements were conducted on a 7.0 T Biospec small animal MRI system (Bruker BioSpin, Germany) with the Paravision 6.0 software platform (https://www.bruker.com/en.html), using a two-element receive-transmit cryogenic quadrature coil. Isoflurane (1.5~2.0%) was used for anesthesia. T_2_-weighted echo-planar imaging sequences were used to acquire MRI data with the following parameters: echo time=35 ms, the field of view=100 mm^2^, acquisition matrix=256 × 256, repetition time=3150 ms, echo train length=8, 28 slices of 0.4 mm. Resting-state functional MRI (rs-fMRI) was performed to assess functional connectivity (FC) between specific regions of interest (ROI) that support numerous cognitive processes: auditory cortex (AC), cingulate cortex (CC), hippocampus (HC), motor cortex (MC), orbitofrontal cortex (OC), somatosensory cortex (SC), thalamus (T), and visual cortex (VC). Rs-fMRI signals were acquired by a T_2_-weighted single-shot echo-planar imaging sequence. The rs-fMRI data analysis was performed using a statistical parametric mapping (SPM) mouse toolbox, which extended the functionality of SPM5 [62]. All selected ROIs were imported into the REST toolbox [63]. FC group comparison between ROIs was tailored software for MATLAB2019, which allowed for *z*-transform by an in-house program and exported of an FC strength correlation matrix. These independently transformed *z*FC-maps were loaded into SPM12 and average *z*FC-maps were analyzed.

### Microbiota methods and measures

Feces of animals were collected after excretion. A total of 190-230 mg of fresh feces were individually collected were frozen in liquid nitrogen immediately. Total bacterial DNA from the feces samples by the EZNA Stool DNA Kit. The concentration, quantity, and purity of the DNA were determined using a TBS-380 mini-fluorometer and NanoDrop 2000 spectrophotometer. The paired-end library and sequencing were constructed by a TruSeq^TM^ DNA Sample Prep Kit. and the Illumina HiSeq4000 platform (Illumina Inc., USA).

The pool of metagenomics data obtained was assembled using MEGAHIT [64]. Contigs with a length of > 300 bp were selected as the final assembling result. MetaGene was then used to predict open reading frames (ORFs) [65]. All predicted genes (> 95% sequence identity) were clustered and mapped to representative sequences by CD-HIT [66] and a short oligonucleotide analysis package [67]. For taxonomic annotations, the representative sequences of the non-redundant genes were aligned to the NCBI NR database (e-value < 1e^-5^) by the BLAST [68].

### Golgi staining

To investigate dendritic spine density, the hemispheres were transferred to the Golgi staining solution. Sample preparation and Golgi staining were performed by FD Rapid Golgistain™ Kits (FD NeuroTechnologies, USA). And then the sections were photographed by a transmission electron microscope (Hitachi, Japan).

### Enzyme-linked immunosorbent assay

The DLG4 / PSD95 ELISA Kit (LifeSpan BioSciences, USA) and synaptophysin ELISA Kit (Lifeome BioLabs, USA) was used to quantify the concentrations of PSD95 and synaptophysin in the hippocampus, respectively. After incubation, the absorbance value was measured at 450 nm by an Enspire™ multilabel reader 2300 (PerkinElmer, Finland).

### Multiplex bead analysis

The hippocampal supernatants were centrifuged, then diluted 1:2 in assay buffer and analyzed by the Luminex 200 (Luminex, USA). Cytokines of the hippocampus including granulocyte colony-stimulating factor (G-CSF), granulocyte-macrophage colony-stimulating factor (GM-CSF), interleukin(IL)-1β, IL-2, IL-4, IL-5, IL-6, IL-17, interferon-γ (IFNγ), interferon-induced protein 10 (IP-10), tumor necrosis factor α (TNF-α), regulated upon activation normal T cell expressed and secreted factor (RANTES), monocyte chemotactic protein-1 (MCP-1), Eotaxin, macrophage inflammatory protein-1β (MIP-1β) were measured by the MILLIPLEX MAP Mouse Cytokine/Chemokine Magnetic Bead Panel (MCYTOMAG-70K, Millipore).

### Statistical analyses

The data were expressed as means ± S.D.. GraphPad Prism8 (GraphPad Software, LLC, USA) was used to statistically analyze and visualize the part data. The comparison of two different groups was performed by Student`s *t*-tests. One-way analysis of variance (ANOVA) followed by Dunnett`s post hoc test or a two-way repeated-measures ANOVA with post-hoc Tukey multiple comparisons test was performed to determine significant differences of data from the multiple groups. The Linear discriminant analysis effect size (LEfSe) statistical analysis was analyzed by the Galaxy Module online, based on *P* < 0.05 and a logarithmic linear discriminant analysis (LDA) score for discriminant features was 3.0 [69]. The correlations between microbial composition and key neurobiological findings were analyzed and plotted by Spearman correlation coefficients (R, version 3.2.4). A principal component analysis (PCA) plot was also generated in R version 3.2.4. Statistical significance was set at *P* <0.05.

### Ethical approval and consent to participate

Animal experiments were processed in accordance with the references in the Guide for the Care and Use of Laboratory Animals published by the National Institutes of Health and were approved by the Institutional Animal Care and Use Committee of Shandong University.
